# Novel bacterial proteolytic and metabolic activity associated with dental erosion-induced oral dysbiosis

**DOI:** 10.1186/s40168-023-01514-0

**Published:** 2023-03-31

**Authors:** Leanne M. Cleaver, Miguel Carda-Diéguez, Rebeca Moazzez, Guy H. Carpenter

**Affiliations:** 1 Centre for Host Microbiome Interactions, Faculty of Dentistry, Oral and Craniofacial Sciences, King’s College London, London, UK; 2 Department of Health & Genomics, Foundation for the Promotion of Health and Biomedical Research (FISABIO) Foundation, Valencia, Spain; 3 Department of Preventive and Restorative Dentistry, Arthur A. Dugoni School of Dentistry, University of The Pacific, San Francisco, USA

**Keywords:** Multi-omics, Salivary microbiome, Dental erosion, Metabolomics, Transcriptomics, Bacterial proteolysis

## Abstract

**Background:**

Dental erosion is a disease of the oral cavity where acids cause a loss of tooth enamel and is defined as having no bacterial involvement. The tooth surface is protected from acid attack by salivary proteins that make up the acquired enamel pellicle (AEP). Bacteria have been shown to readily degrade salivary proteins, and some of which are present in the AEP. This study aimed to explore the role of bacteria in dental erosion using a multi-omics approach by comparing saliva collected from participants with dental erosion and healthy controls.

**Results:**

Salivary proteomics was assessed by liquid-chromatography mass spectrometry (LC–MS) and demonstrated two altered AEP proteins in erosion, prolactin inducible protein (PIP), and zinc-alpha-2 glycoprotein (ZAG). Immunoblotting further suggested that degradation of PIP and ZAG is associated with erosion.

Salivary microbiome analysis was performed by sequencing the bacterial 16S rRNA gene (V1-V2 region, Illumina) and showed that participants with dental erosion had a significantly (*p* < 0.05) less diverse microbiome than healthy controls (observed and Shannon diversity). Sequencing of bacterial mRNA for gene expression (Illumina sequencing) demonstrated that genes over-expressed in saliva from erosion participants included H + proton transporter genes, and three protease genes (*msrAB*,* vanY*, and *ppdC*). Salivary metabolomics was assessed using nuclear magnetic resonance spectrometry (NMR). Metabolite concentrations correlated with gene expression, demonstrating that the dental erosion group had strong correlations between metabolites associated with protein degradation and amino acid fermentation.

**Conclusions:**

We conclude that microbial proteolysis of salivary proteins found in the protective acquired enamel pellicle strongly correlates with dental erosion, and we propose four novel microbial genes implicated in this process.

Video Abstract

**Supplementary Information:**

The online version contains supplementary material available at 10.1186/s40168-023-01514-0.

## Introduction

Dental erosion is a common disease with an estimated prevalence of approximately 30% worldwide in children, adolescents, and adults [1–3]. Whilst dental erosion has been classified as loss of tooth enamel and has been associated with increased consumption of citrus fruits, soft drinks, sports drinks, and gastro-oesophageal reflux disease (GORD) [4], its underlying mechanism is poorly understood. Despite it being a common disease of the oral cavity, which harbors the body’s second largest microbiome, it is defined as having no bacterial involvement, which is one factor distinguishing it from dental caries [5].

The tooth surface is covered by a subset of salivary proteins known as the acquired enamel pellicle (AEP) to which bacteria can bind reversibly (reviewed by [6]) and form biofilms. The AEP offers a level of protection to the enamel surface [7–10] from dietary and esophageal acids, and is a modulator of dental erosion and erosive tooth wear progression. The binding of early colonizers to glycan motifs and sialic acids on salivary proteins adsorbed onto the tooth surface has been known for some time [11]. There have been over 360 proteins identified in the AEP including alpha-amylase, mucin 5b, lysozyme, histones, cystatin, zinc-alpha-2-glycoprotein and statherin [12–14]. Small peptides are also present in the AEP derived from salivary proteins, the majority of which are negatively charged and hydrophobic, suggesting proteolytic activity occurs within the AEP [15].

Some proteins within the AEP have been shown to be susceptible to bacterial degradation in saliva, such as histatin and statherin. Salivary pellicles that were assessed after a 2-h and 24-h formation when participants were fasting showed a decreased maturation of the AEP and decreased amino acid composition of the AEP, suggesting that bacteria may degrade the pellicle in periods of starvation [16]. There are significantly reduced amounts of pellicle proteins in individuals with dental erosion compared to healthy controls [17], in particular a reduction in statherin [18], which again suggests the presence of proteases affecting the pellicle. Studies of this type are challenging, and assessing the proteolytic degradation of the AEP over time is difficult to achieve both in vivo and in vitro.

Bacteria have been shown previously to readily degrade salivary proteins [19, 20] through glycosidase, protease, and peptidase activity. Commensal bacteria *Streptococcus mitis* and *S. mutans* have been shown previously to exploit mucins and lower molecular weight salivary proteins as a nutrient source and also for binding and colonization of the oral cavity [21]. *Lactobacillus fermentum*, another important oral commensal, has also been shown to degrade salivary mucin 5b for adherence and as a nutrient source [22]. As AEP is difficult to study, and saliva is subject to proteolytic degradation by bacteria, it was chosen here as a suitable alternative for multiple analyses. 

Despite the plethora of microbiome analyses in dental caries, periodontitis, and obesity, to name a few, there have been limited investigations into the role of microbial dysbiosis in dental erosion. A simple search of ‘dental erosion microbiome’ results in only one piece of peer-reviewed published work that details the salivary microbiome in patients with GORD [23]. It has been shown that the pH of the oral cavity, in particular the teeth (when tested using antimony electrodes), can remain at a lower pH (< 4) for long after the consumption of a carbonated drink in patients with erosion compared to healthy controls [24, 25]. Therefore, those patients with dental erosion that have a regular and habitual intake of acidic food and drink may have a distinctly different oral microbiome compared to those without dental erosion due to an overall more acidic growth environment.

As with the microbiome analysis of people who suffer from dental erosion, there has been limited investigation of the metabolomics of dental erosion. It is understood that in dental erosion, the acids present in the oral cavity are not associated with producing organic acids by cariogenic bacteria, but derive mainly from dietary consumption. Therefore, this current study was interested in assessing whether the metabolomics of individuals with dental erosion was different from healthy individuals.

Since oral bacteria can degrade salivary proteins by protease and peptidase activity, and produce organic acids from amino acid fermentation, their role in dental erosion was investigated here using a multi-omics approach.

## Materials and methods

### Part A: previously collected saliva samples (*n* = 30 erosion, *n* = 30 healthy controls)

**Fig. 1:**
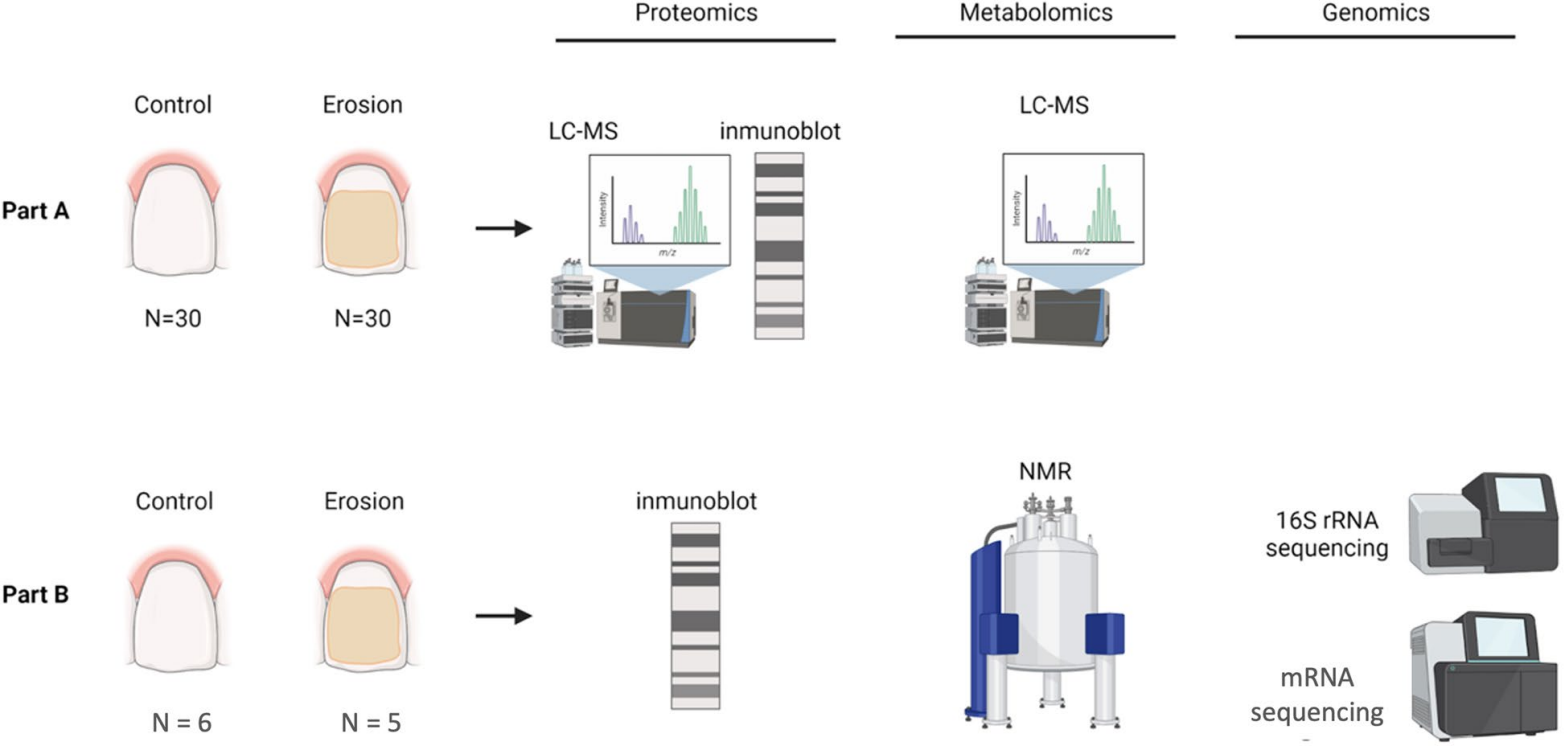
Graphical representation of methods performed on Part A (top) and Part B (bottom) saliva samples from erosion and control participants. Figure was created using https://BioRender.com

Saliva samples analyzed in the first part of this study were collected for a previous study. Details of their collection are noted in [9, 17] Ethical approval (NHS No. 10/H0703/11), and informed consent was obtained from participants aged between 18 and 65 years. Participants underwent an oral examination to determine their basic erosive wear examination (BEWE) score [26] and to perform a basic examination of the soft and hard tissues. Participants with evidence of dental erosion with a BEWE score of greater than 8 extant cumulative score with one score of 3 in a sextant were assigned to the erosion group, and participants that showed no evidence of dental erosion with a BEWE score ≤ 8 per extant cumulative score and scores of less than 3 in all sextants were assigned to the healthy group (Fig. 1 Part A).

Resting, unstimulated whole mouth saliva was collected by expectorating for 5 min and samples were stored after collection at − 70 °C.

### Part B: newly collected saliva samples (*n* = 5 erosion, *n* = 6 healthy controls)

Ethical approval was granted for this study by the Northampton Research Ethics Council under REC reference: Northampton REC, 14/EM/0183, and informed consent was obtained from all participants aged between 18 and 75 years. Participants were invited to participate in the study if they were in good general health, with no diseases of the hard and soft tissues in the mouth, no active caries, and had not taken antimicrobials within 3 months of inclusion in this study. Again, participants were split into two groups based on their BEWE score [26] in the same manner as Part A. Stimulated whole mouth saliva was collected by participants chewing on a piece of flavorless parafilm and expectorating approximately 6 ml saliva (Fig. 1 Part B).

In both instances, participants refrained from eating or drinking anything, other than water, for at least an hour before saliva collection. Saliva was collected in the afternoon.

#### Salivary proteomics

Using samples from Part A, ten randomly selected dental erosion and ten randomly selected healthy control saliva samples were assessed by liquid chromatography mass spectrometry (LC–MS) by the Proteomics Facility, Denmark Hill, King’s College London. Briefly, saliva samples containing 125 μg protein were prepared in a stacking gel, denatured, trypsin-digested and labeled with TMTsixplex (Thermo Fisher Scientific) isobaric quantitation labels. The samples were then combined in 4 groups of five with the sixth sample being a pooled saliva reference. Samples were analyzed by Orbitrap (Thermo Fisher Scientific) LC–MS, and the resulting peptides were identified using Scaffold (version 4.3.0). The mass spectrometry proteomics data have been deposited to the ProteomeXchange Consortium via the PRIDE [27] partner repository with the dataset identifier PXD035565 and 10.6019/PXD035565.

Based on results from LC–MS analysis, saliva samples were analysed for proteolytic degradation by immuno-blotting saliva samples from both studies (Parts A and B) for prolactin inducible protein (PIP) and zinc-alpha-2 glycoprotein (ZAG) (in triplicate). Samples were centrifuged for 2 min at 13,000 RPM to remove cell debris, and equal volumes of samples were separated by running samples on a 4–12% Bis–Tris gel (Life Technologies), along with SeeBlue Plus2 pre-stained protein standard (Thermo Fisher Scientific). Proteins were transferred onto nitrocellulose membranes and washed with Tris-buffered saline plus Tween 20 (TBST). Primary antibodies were prepared in TBST plus 5% skim milk powder (Sigma-Aldrich) (rabbit anti-PIP 1:2000, mouse anti-ZAG 1:2500), and the membranes were incubated at room temperature on a rotary rocker for one hour. Membranes were washed with TBST prior to adding horseradish peroxidase (HRP) labeled secondary antibodies (goat anti-mouse HRP antibody for anti-PIP and goat anti-rabbit HRP antibody for anti-ZAG 1:5000) for a further 1-h incubation on a rotary rocker at room temperature. The blots were washed with TBST prior to chemiluminescence immunodetection of HRP conjugated secondary antibodies visualized using the ChemiDoc Imaging System (Bio-Rad).

#### Salivary amino acid concentration

Twenty randomly chosen samples per group from Part A were assessed for salivary amino acid concentration using mass spectrometry as described in the previous salivary proteomics section. Amino acid concentrations were reported in μmol/L. The mean concentration was reported for each group.

#### Salivary metabolomics

For the subsequent study (Part B), nuclear magnetic resonance (NMR) analysis on thawed saliva aliquots (500 μl, *n* = 3 per participant, 5 erosion and 6 healthy controls) was performed using a previously published method [19]. Briefly, samples were centrifuged for 5 min at 13,000 RPM to remove cell debris. The supernatant was removed for NMR analysis and mixed at a ratio of 4:1 with sodium trimethylsilyl propionate (TSP) buffer and added to a 5 mm NMR tube (Bruker, Coventry, UK). Spectra were acquired on a 600 MHz NMR spectrophotometer (Bruker). The spectra were processed on TopSpin (Bruker) to correct the phase and baseline and to calibrate the TSP peak to 0 ppm. The identity of metabolites were assigned and the concentration (mM) obtained from spectra using Chenomx NMR Suite version 8.5 (Chenomix Ltd., Canada). This data is available at the NIH Common Fund’s National Metabolomics Data Repository (NMDR) website, the Metabolomics Workbench, https://www.metabolomicsworkbench.org, where it has been assigned Project ID PR001447. The data can be accessed directly via its Project DOI: 10.21228/M8PH7X.

#### Salivary bacterial 16S rRNA gene sequencing

One milliliter of each participant’s saliva (Part B only) was centrifuged (13,000 RPM for 3 min). The supernatant was removed from the pellet, and bacterial DNA was extracted from pellets using the GenElute™ Bacterial Genomic DNA Kit (Sigma-Aldrich, Dorset, UK) according to the manufacturer’s instructions with the addition of a lysozyme (Sigma-Aldrich) lysis step for 30 min at 37 °C to aid in the lysis of Gram-positive organisms. Bacterial DNA was eluted into 200 μl elution buffer.

The variable regions V1-V2 of the 16S rRNA gene were amplified by polymerase chain reaction (PCR) using a previously published method [28] using 27F (with YM modifications) and 338R primers with Illumina (Illumina, Cambridge, UK) barcodes and adapters, amplification products were purified using the QiaQuick PCR Purification kit (Qiagen, Manchester, UK), and libraries were pooled for sequencing at a concentration of 10 nM. Samples were sequenced at the Queen Mary University of London Genome Centre using the Illumina MiSeq (Illumina) platform v3 2 × 250 bp flow cell for paired-end sequencing. Sequences were obtained in fastq format and were analyzed using the dada2 pipeline [29] (version 1.21.0) in R (version 4.2.2) and RStudio (version 1.4.1717), sequences were assigned genus/species using the Human Oral Microbiome Database (HOMD; version 15.9) 16S rRNA database, and alpha and beta diversity was calculated using R (version 4.2.2) and RStudio (version 1.4.1717). Packages used were phyloseq [30], ggplot2 [31], readr, plyr [32], reshape2 [33], RColorBrewer, ggpubr, and vegan [34]. Read abundance was normalized using ANCOMBC [35]. Wilcoxon test was used to compare the normalized abundance between groups and the *p* values were adjusted using false discovery rate method [36]. In addition to ANCOMBC, Boruta test was used to confirm relevant differential features [37]. The raw sequence reads (fastq) have been deposited in the NCBI Sequence Read Archive with the BioProject ID PRJNA866346.

#### Salivary bacterial transcriptomics

Another 1 ml of participant’s saliva (Part B only) was centrifuged as above, the supernatant was removed, and the bacterial RNA from the pellet was purified using the Quick-RNA Fungal/Bacterial Microprep Kit (Zymo Research, Cambridge, UK) according to the manufacturer’s protocol, with an on-column DNase I digestion (Qiagen). RNA was eluted into 15 μl RNase-free molecular grade water provided with the kit. The quality of bacterial RNA from each sample was assessed using the Agilent 2100 Bioanalyzer Prokaryote Total RNA Pico (Agilent Technologies, Cheadle, UK), and bacterial rRNA was depleted using the NEBNext rRNA depletion kit (bacterial; New England BioLabs, Hitchin, UK) according to the manufacturer’s protocol at King’s College London Genome Centre, UK. RNA libraries were prepared using the NEBNext Ultra II Directional RNA library prep kit for Illumina (New England BioLabs), plus the NEBNext Multiplex Oligos for Illumina (index primer sets 1 and 2; New England BioLabs) following the manufacturer’s protocol with slight modifications to fragmentation time based on RNA integrity number (RIN; RIN > 6 = 15-min fragmentation (5 out of 11), RIN 2–6 = 8 min fragmentation (6 out of 11)). Libraries were pooled and sent to GeneWiz (Azenta Life Sciences, Essex, UK) and sequenced using the Illumina HiSeq (Illumina) platform 2 × 150 bp flow cell to obtain a depth of 8–9 million reads per sample. RNA sequence data was received in fasta file format and was analyzed using a previously published methodology [38] using R (version 4.2.2) and RStudio (version 1.4.1717) at the FISABIO Research Institute, Valencia, Spain. Briefly, reads were trimmed per quality and length, then host and ribosomal reads were removed, and the remaining reads were aligned and annotated taxonomically and functionally using Bowtie2 (version 2.4.5) to a curated version of the HOMD database (see Carda-Diéguez et al. 2022). KEGG annotations in Table 1 were manually revised using BLASTP against NCBI and UNIPROT databases. The number of reads were normalized to the 16S sequencing data and compared between groups following DESeq2 method [39] using Wald testing. The raw sequence reads (fastq) have been deposited in the NCBI Sequence Read Archive with the BioProject ID PRJNA866346.

**Table 1:** Bacterial genes upregulated in the erosion group samples (log2FC; log twofold change, %; percentage relative abundance, *p* adjusted < 0.05)

**log2FC**	**Healthy(%)**	**Erosion (%)**	**Gene**	**Gene function**	**Curated annotation**
1.06	3.84E − 02	8.02E − 02	msrAB; peptide methionine sulfoxide reductase msrA/msrB [EC:1.8.4.11 1.8.4.12]	Catalyses methionine sulfoxide in proteins into methionine	
1.26	6.81E − 03	1.63E − 02	efeO; iron uptake system component EfeO	Iron uptake	
1.32	2.56E − 02	6.38E − 02	vanY; zinc D-Ala-D-Ala carboxypeptidase [EC:3.4.17.14]	Bacterial cell wall metabolism metallopeptidase	LD-carboxypeptidase LdcB/DacB
1.33	3.57E − 03	8.95E − 03	kinB; two-component system, sporulation sensor kinase B [EC:2.7.13.3]	Alginate biosynthesis	Putative membrane protein
1.37	1.74E − 02	4.51E − 02	exoZ; exopolysaccharide production protein ExoZ	Acetyltransferase	Acetyltransferase
1.38	8.72E − 05	2.27E − 04	ttdA; L( +)-tartrate dehydratase alpha subunit [EC:4.2.1.32]	Transcription factor	
1.51	1.23E − 02	3.50E − 02	mprF, fmtC; phosphatidylglycerol lysyltransferase [EC:2.3.2.3]	Multiple peptide resistance factor	
1.54	5.52E − 04	1.60E − 03	EPHX1; microsomal epoxide hydrolase [EC:3.3.2.9]	Microsomal epoxide hydrolase	
1.66	1.12E − 04	3.54E − 04	uspB; universal stress protein B	Ethanol resistance gene	Helicase PriA essential for oriC/DnaA-independent DNA replication
1.69	1.17E − 03	3.77E − 03	kefF; glutathione-regulated potassium-efflux system ancillary protein KefF	Potassium efflux	
1.70	1.14E − 02	3.71E − 02	poxB; pyruvate dehydrogenase (quinone) [EC:1.2.5.1]	Cell membrane enzyme	
1.81	3.62E − 02	1.27E − 01	ME2, sfcA, maeA; malate dehydrogenase (oxaloacetate-decarboxylating) [EC:1.1.1.38]	Malic enzyme	
1.86	2.40E − 03	8.69E − 03	mpaA; protein MpaA	Peptidoglycan degradation	LPXTG cell wall surface protein, zinc carboxypeptidase family
1.97	1.27E − 04	4.98E − 04	lcdH, cdhA; carnitine 3-dehydrogenase [EC:1.1.1.108]	Oxidation of L-carnitine to 3-dehydrocarnitine	Cell division protein FtsQ
1.97	9.30E − 05	3.66E − 04	K09964; uncharacterized protein	Uncharacterised	
2.02	3.26E − 02	1.32E − 01	K07396; putative protein-disulfide isomerase	Putative protein-disulfide isomerase	DsbA family oxidoreductase
2.07	4.14E − 03	1.74E − 02	uctC; CoA:oxalate CoA-transferase [EC:2.8.3.19]	Oxalate CoA-transferase	
2.17	9.16E − 03	4.11E − 02	traG; conjugal transfer mating pair stabilization protein TraG	NTP hydrolases for bacterial conjugation	MucBP domain-containing protein
2.17	7.43E − 04	3.34E − 03	salR; two-component system, NarL family, secretion system response regulator SalR	Nitrate regulatory gene	
2.18	6.68E − 04	3.03E − 03	gluB; glutamate transport system substrate-binding protein	Glutamate transport system substrate-binding protein	
2.19	9.53E − 04	4.35E − 03	raxB, cvaB; ATP-binding cassette, subfamily B, bacterial RaxB /	Adenylate cyclase and type I secretion system	Peptidase domain-containing ABC transporter
2.20	1.75E − 05	8.22E − 05	lrgA; holin-like protein	Inhibits the expression or activity of extracellular murein hydrolases	
2.22	5.04E − 04	2.35E − 03	ACADL; long-chain-acyl-CoA dehydrogenase [EC:1.3.8.8]	Long-chain-acyl-CoA dehydrogenase	
2.30	3.31E − 04	1.63E − 03	ppdC; prepilin peptidase dependent protein C /unknown function	Pilus biogenesis	
2.43	1.87E − 04	1.01E − 03	ACR3, arsB; arsenite transporter	Arsenic resistance system, transporter	Citrate transporter
2.67	6.56E − 04	4.19E − 03	adaA; AraC family transcriptional regulator, regulatory protein of adaptative response	Transcriptional regulator	Phosphoribosylaminoimidazole-succinocarboxamide synthase, 6.3.2.6, SAICAR synthetase
2.73	3.27E − 05	2.21E-04	adiC; arginine:agmatine antiporter	Arginine dependent acid resistance	PrgI family protein
3.25	3.15E − 04	3.00E − 03	pksJ; polyketide synthase PksJ	Involved in the pathway bacillaene biosynthesis	Non-ribosomal peptide synthetase
3.33	1.25E − 04	1.26E − 03	K09388; uncharacterized protein	Uncharacterised	
3.85	4.64E − 05	6.77E − 04	pscB; photosystem P840 reaction center iron-sulfur protein	Bacterial inmunity	Type I-E CRISPR-associated protein Cas7/Cse4/CasC
3.96	7.43E − 05	1.17E − 03	atuB; citronellol/citronellal dehydrogenase	Citronellol/citronellal dehydrogenase	Amino acid transporter
4.17	5.93E − 06	1.15E − 04	K09120; uncharacterized protein	Uncharacterised	
4.21	2.29E − 04	4.25E − 03	PMA1, PMA2; H + -transporting ATPase [EC:7.1.2.1]	Active transport of protons	ABC transporter ATP-binding protein

## Results

### Salivary proteomics

The LC–MS targeted proteomic analysis of ten randomly selected dental erosion and ten randomly selected healthy control saliva samples from Part A identified 160 salivary proteins, with seven of these salivary proteins showing significant differences between the two groups when analyzed by *t* test (*p* < 0.05). These included mucin 5B, lactoperoxidase, human UPF0762, Ig kappa chain C region, serotransferrin, prolactin-inducible protein (PIP), and zinc-alpha-2 glycoprotein (ZAG). As PIP and ZAG are proteins that are present in the acquired enamel pellicle, these were investigated further by immunoblotting.

**Fig. 2:**
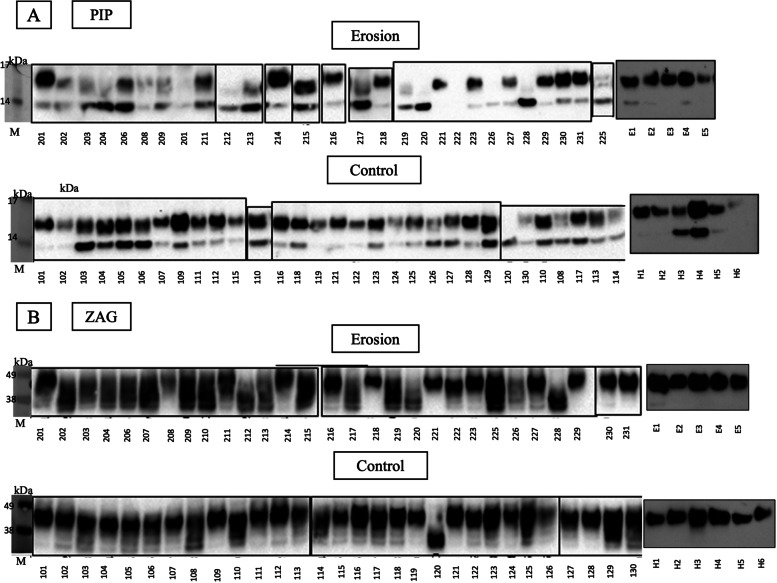
Immunoblots for (**A**) PIP and (**B**) ZAG proteins identified in saliva samples (E = erosion, H = healthy controls, M = protein marker)

Figure 2 shows immunoblots for PIP and ZAG from both studies. In addition to the expected 15 kDa PIP protein band in all but 2 control subjects (120 and H6), an additional smaller 12 kDa cleavage product can be seen in Fig. 2 (A). The blots revealed mostly intact PIP in saliva from controls (upper band), but in erosion samples, there was a distinct degradation of the upper band in 17 out of 33 subjects (51%). Similarly, immunoprobing saliva samples for ZAG demonstrated an expected protein band at 42 kDa and evidence of degradation of ZAG (Fig. 2 (B)). Although there were multiple lower molecular weight bands in both the control samples and erosion samples, more erosion samples showed a loss of the upper band; 16 out of 34 (47%) erosion samples had a degraded upper band compared to 5 out of 36 (14%) of healthy control samples.

All saliva samples from Part A were also assessed for mammalian protease activity using elastase, collagenase, and gelatinase assays (elastase and collagenase activity shown in Supplementary Fig. 1, no significant difference in mean values of each group when tested by t-test); however, there were no correlations with PIP and ZAG degradation.

### Salivary amino acids

**Fig. 3:**
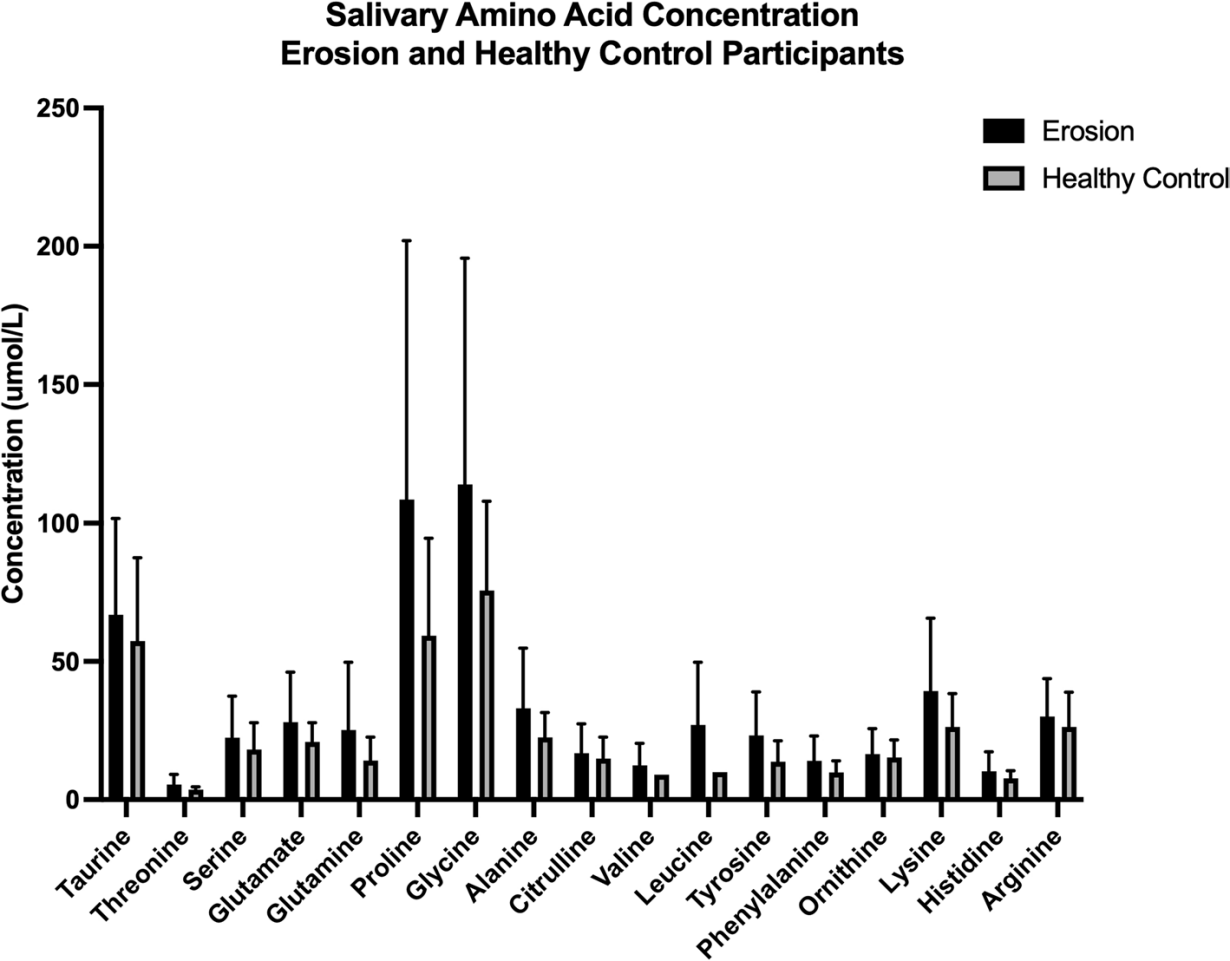
Salivary amino acid concentration erosion and healthy control participants. Salivary amino acid composition and concentration in erosion (black bars, *n* = 20) and healthy control (gray bars, *n* = 20) saliva samples (mean ± SD)

The initial study (Part A) assessing amino acid composition and concentration of saliva samples (*n* = 1 per participant, 20 randomly chosen samples for each group) using mass spectrometry can be seen in Fig. 3. A total of 19 amino acids were identified. The data was not normally distributed when tested by Shapiro-Wilks normality; therefore, Wilcoxon ranked paired *t* test was performed on the whole data set which demonstrated a significant difference (*p* < 0.0001) between the erosion and healthy control group. Although not significant, there was a trend for increased proline and glycine concentrations in the erosion group.

### Salivary microbiome composition

**Fig. 4:**
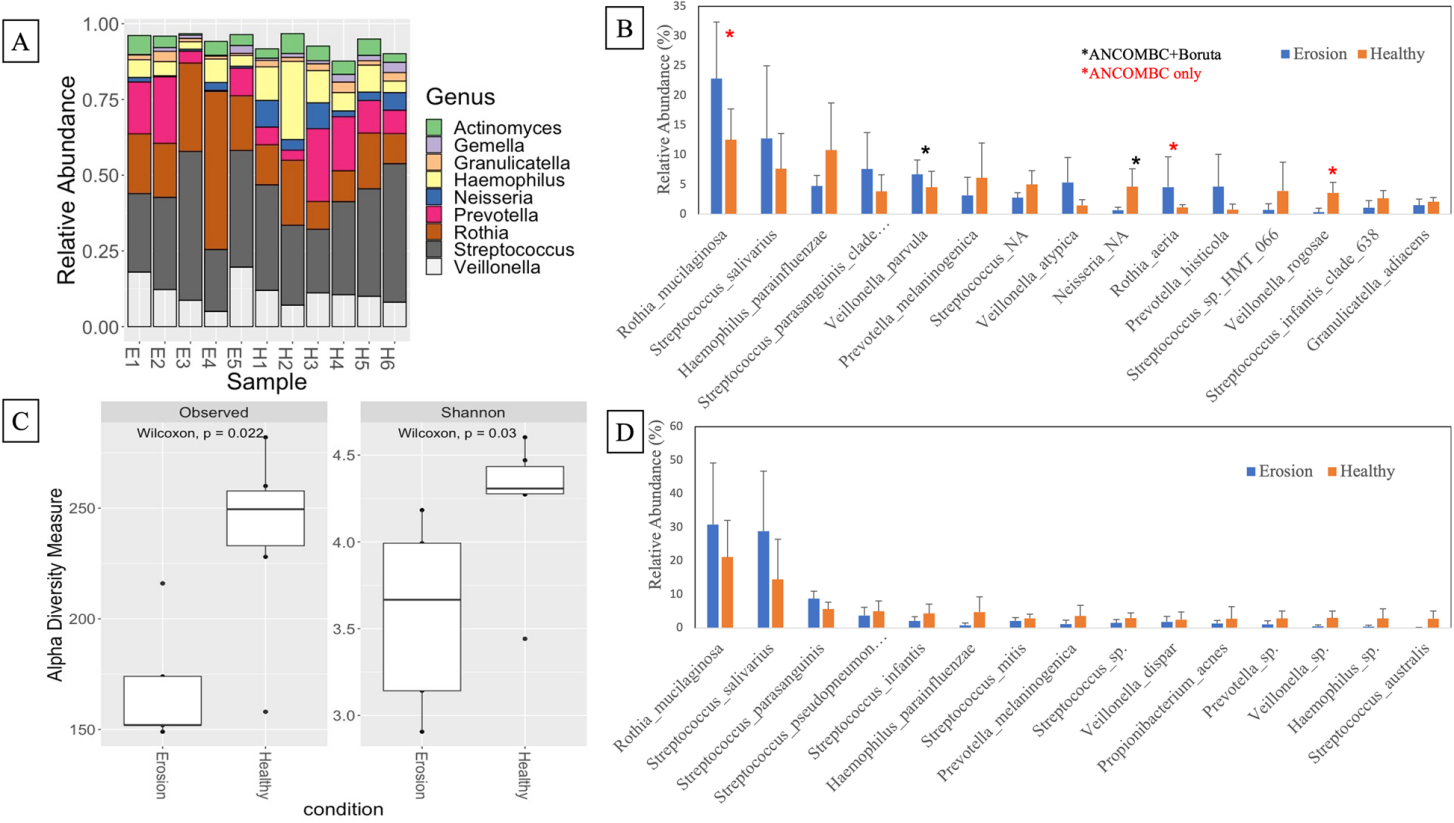
**A** Relative abundance of the top 9 genera in individual saliva samples from participants with and without erosion (E; erosion, H; healthy), **B** relative abundance of individual species in erosion (blue) and healthy (orange) samples that were statistically significantly (ANCOMBC + Boruta) different (black asterix) and ANCOM-only differing species (red asterix), **C** observed and Shannon alpha diversity of gene sequences in erosion samples compared to healthy control samples, and **D** relative abundance of transcriptomically active bacterial species in erosion (blue) and healthy (orange) saliva samples

All saliva samples were sequenced successfully, and after quality filtering and removal of chimeras, there were 425,064 sequences available for analysis (between 32,000 and 44,000 sequences per sample). The relative abundance of the top 9 genera in individual participant samples can be seen in Fig. 4 (A), which suggests that individuals with erosion have higher relative abundance of *Rothia* and a decreased relative abundance of *Neisseria*. After analysis using ANCOMBC normalization and Boruta test to confirm the differential features, *Veillonella parvula* was significantly (*p* < 0.005) more abundant in erosion samples, and unclassified *Neisseria sp*. was significantly (*p* < 0.0005) more abundant in the healthy samples (Fig. 4 (B)). When using ANCOMBC alone, there was a tendency (adjusted *p* value < 0.05) for increased abundance of *Rothia mucilaginosa* and *Rothia aeria* in erosion samples, and a tendency for increased abundance of *Veillonella rogosae* in healthy samples. Other lower abundant species that were significantly increased in erosion samples compared to healthy (not included in Fig. 4 (B)) were *Streptococcus mutans* (*p* < 0.0001) *Actinomyces species HMT 169* (*p* < 0.005), *Streptococcus species HMT 064* (*p* < 0.005), and the lower abundant species that were significantly increased in healthy control samples compared to erosion were *Peptostreptococcus stomatis* (*p* < 0.0001), *Fusobacterium periodonticum* (*p* < 0.001), *Peptostreptococcaceae sulci* (*p* < 0.0001), *Mogibacterium diversum* (*p* < 0.0001), and *Oribacterium parvum* (*p* < 0.0001).

Alpha diversity between the two groups was calculated using Wilcoxon ranked test, which showed a statistically significant decrease in salivary microbiome diversity (Observed and Shannon) in erosion participant samples compared to healthy samples (*p* < 0.05, Fig. 4 (C)). Beta diversity between the two groups was calculated using Bray–Curtis dissimilarity matrix permutational multivariate analysis of variance (PERMANOVA) testing, which demonstrated a significant difference between the two groups (*p* 0.009), and non-metric multidimensional scaling (NDMS), which returned an NDMS stress of 0.07982286 (Supplementary Fig. 2), further demonstrating the significant difference in microbial abundances between the two groups.

### Salivary transcriptomics

Saliva RNA samples were sequenced to achieve greater than 10 million reads per sample. After quality filtering and host and ribosomal RNA removal (rarefaction curves in Supplementary Fig. 3), there were 4 ± 1 × 10^7^ reads per sample available for annotation and analysis. Using a curated version of the HOMD database, we were able to map 78.5 ± 7% of the reads to the database. Approximately 6700 genes were identified in the eleven samples analyzed using DESeq2. Figure 4 (D) shows the relative abundance (%) of mRNA sequences assigned to bacterial species within the samples. When assessing Fig. 4 (B) and (D) together, the most abundant bacterial species in the samples were also the most active (*Rothia mucilaginosa* and *Streptococcus salivarius*) as they had the greatest number of relative abundant genes in the samples.

**Fig. 5:**
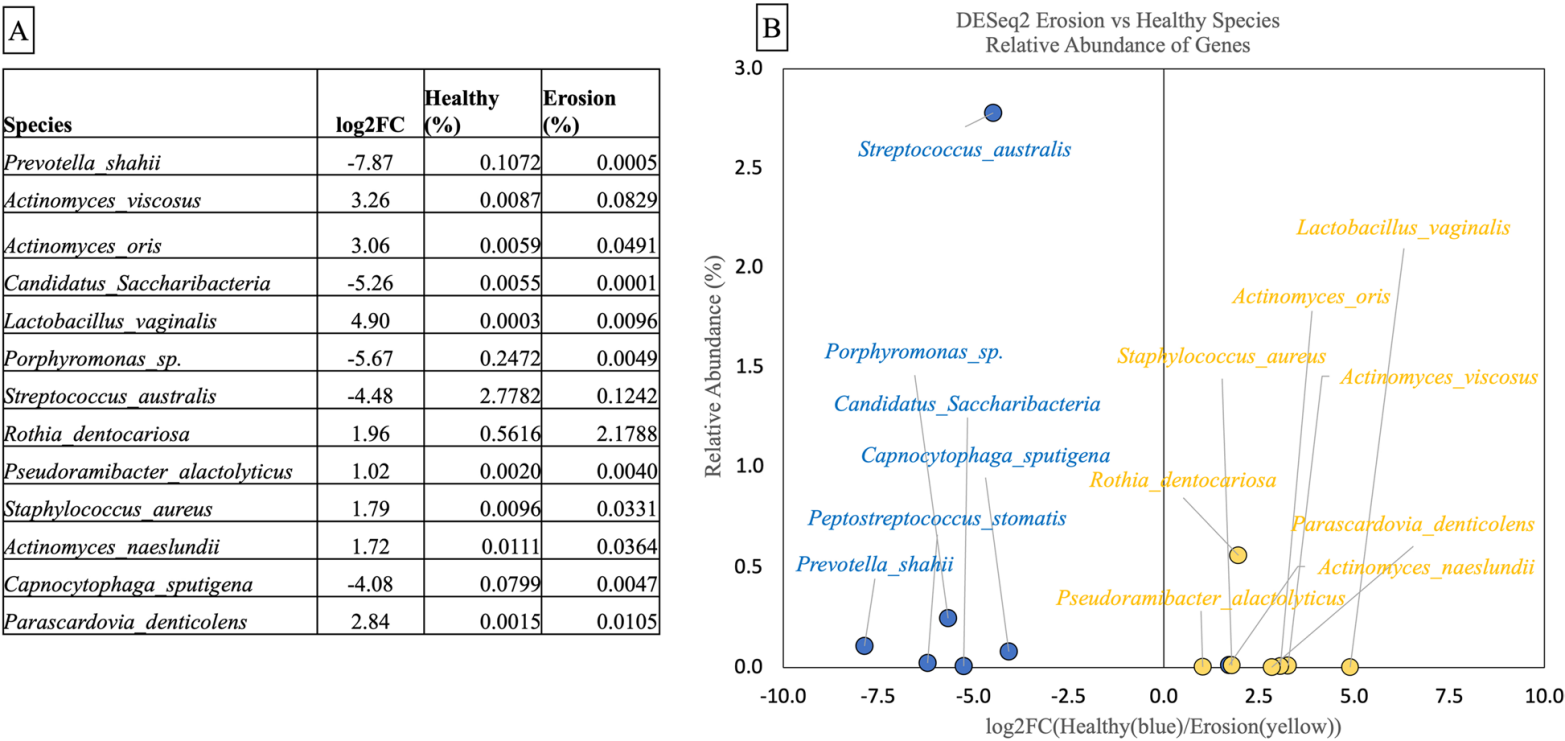
**A** Bacterial species that were statistically differentially represented in mRNA sequencing data (mean of the sample groups) between erosion and healthy group samples; therefore, more active in erosion or healthy participants (mean relative abundance, %). Species with a negative log2FC value represent species that are differentially more active in healthy samples, positive values represent species that are differentially more active in erosion samples. **B** The same data shown graphically (healthy in blue, erosion in yellow)

DESeq2 was employed to characterise bacterial species across the two groups that were significantly (*p* < 0.05) differentially active by the abundance of mRNA assigned to genes. Figure 5 (A) demonstrates the relative abundance (%) of species in healthy and erosion groups and the log twofold change of these species between the two groups, and Fig. 5 (B) shows this schematically. The most differentially significant active species in erosion were *Rothia denticariosa*, *Actinomyces viscosus*, *oris* and *naeslundii*, *Lactobacillus vaginalis*, *Staphylococcus aureus, Pseudoramibacter alactolyticus*, and *Pasascardovia denticolens*. The most differentially significant active species in healthy controls were *Prevotella shahii*, *Candidatus Saccharibacteria*, *Porphyromonas species*,* Streptococcus australis*, and *Capnocytophaga sputigena*. These species were significantly more active in these sample groups, yet their relative abundance in the samples is low.

Bacterial genes over-expressed in the erosion group can be seen in Table 1. Two genes which were highly over-expressed and therefore of particular interest were the arginine:agmatine antiporter gene (*adjC*) and the H + -transporting ATPase gene (*PMA1/PMA2*). There were three protease genes over-expressed in the erosion group; *msrAB*, which is a protease that catalyses methionine sulfoxide into methionine, *vanY*, which is a protease involved in bacterial cell wall metabolism, and *ppdC*, which is a protease required in pilus biogenesis.

### Metabolite and transcriptome correlations

**Fig. 6:**
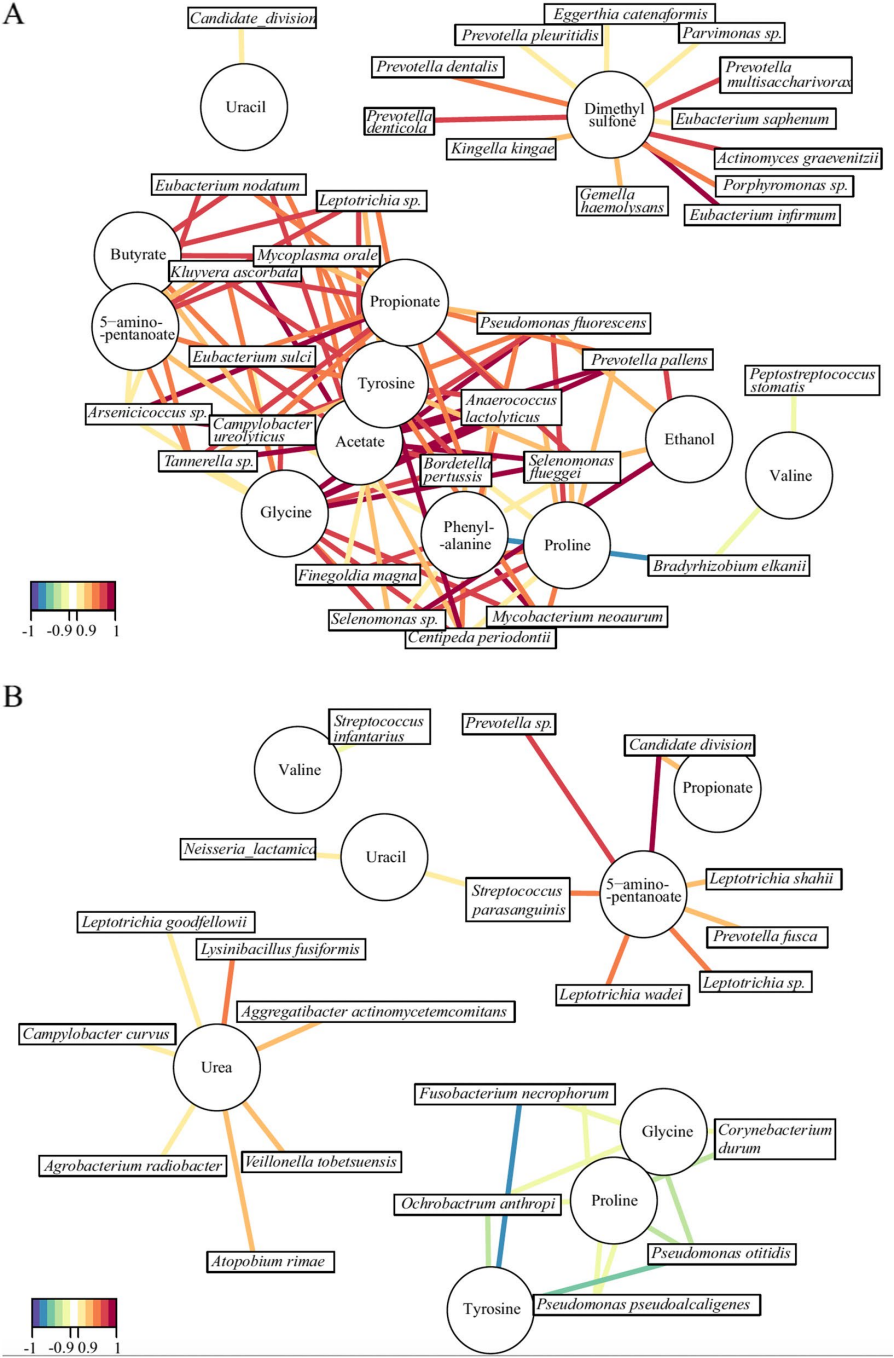
Bacteria-salivary metabolite correlation networks in erosion (**A**) and healthy (**B**) mRNA sequencing samples. Positive and negative significant correlations between individual species (squares) and metabolites (circles) are represented with coloured lines according to the key (red is positive correlation). Metabolites were identified by NMR analysis

Untargeted metabolomics was performed on all saliva samples (Part B) in triplicate using nuclear magnetic resonance (NMR) spectrometry. When the metabolite concentration data was analysed as stand-alone results without correlation, there was no significant difference between erosion and healthy control participants (*p* > 0.05, multiple Mann–Whitney tests, Supplementary Fig. 4). However, when concentrations of metabolites correlated with mRNA sequencing data (Part B) to establish any connections between bacterial species abundance and metabolite profiles for both groups there were more bacterial species positively correlated with common metabolites in the erosion group compared to the healthy control group (Fig. 6). The dental erosion group had strong correlations between metabolites associated with protein degradation and amino acid fermentation (formate, butyrate, propionate, 5-aminopentanoate, acetate, glycine, phenylalanine, dimethyl sulfone) and increased activity of species including 4 *Prevotella* species, *Actinomyces graevenitzii*, *Tannerella species*, and 2 *Selenenomas* species, despite these species not being of high abundance in the samples. Whereas in the healthy control group, the only positive correlations between metabolite concentrations and bacterial activity were for urea and 5-aminopentanoate; urea was positively correlated with *Aggregatibacter actinomycetecomytans*, *Lysinibacillus fusiformis*, and *Veillonella tobetsuensis*, and 5-aminopentanoate was positively correlated with 3 different *Leptotrichia species*, *Streptococcus parasanguinis*, and 2 *Prevotella species.* The gene expression data and metabolomic data was also correlated with the Kyoto Encyclopaedia of Genes and Genomes (KEGG) database (see Supplementary Fig. 5A (erosion) and 5B (healthy)). Interestingly, when bacterial gene expression was correlated with metabolite concentration, only urea showed significant correlations in healthy samples, which was also observed when metabolites and species abundance was correlated.

## Discussion

This preliminary study is the first to demonstrate a difference in salivary protein degradation, salivary microbiome composition, and salivary metabolite profiles between individuals with dental erosion and healthy controls using an integrated multi-omics approach.

The initial study conducted here (Part A) clearly showed significant changes in two AEP-associated salivary proteins, PIP and ZAG, and increased free amino acids in saliva by mass spectrometry. Assays for mammalian proteases showed no correlation to salivary degradation, which suggested microbial proteolytic activity. Immunoblotting of all saliva samples further demonstrated proteolytic changes in these salivary proteins, with erosion participants demonstrating more salivary protein degradation than healthy controls. In the subsequent study (Part B), there were more bacterial species that positively correlated with the identified metabolites in the dental erosion group, including metabolites that have previously been associated with proteolytic degradation. This suggests that, whilst the metabolites are similar in concentration, there are more, and significantly different species of, bacteria present in the erosion group that are capable of degrading and utilizing different subtrates, including salivary proteins that are present in the AEP. As the metatranscriptomics portion of this study is limited by sample size, the results require corroboration with a higher number of participants in each group.

The identification of several bacterial proteases (*msrAB*, *vanY* and *ppdC*, see Table 1) over-expressed in the erosion group also suggested proteolytic activity in saliva from subjects with erosion. We have shown previously that the presence of proline, glycine, 5-aminopentanoate, butyrate, acetate, and propionate are all metabolic markers of increased bacterial proteolysis [19, 40]. This study may also suggest that PIP and ZAG degradation are associated with the presence of *Prevotella* in the oral cavity which may be a candidate species to degrade these proteins, as this genus was seen in higher abundance in PIP and/or ZAG degraders (not significant, data not shown). *Prevotella nigrescens* and *intermedia* have been shown previously to degrade proteins in the oral cavity [41], but in relation to dental abscesses and the cleavage of proteins associated with gingival crevicular fluid. *Prevotella nigrescens* has also been associated with protein degradation in periodontitis [42], increasing expression of metalloproteases as a virulence factor in this species. None of the participants included in this study had any evidence of periodontal disease.

Although the data in this paper is only associative and not causal, and particularly because the data assessed salivary protein degradation and not AEP protein degradation, it is tempting to speculate that if the proteins within the protective AEP were compromised by microbial proteolytic activity this could be a contributing factor to dental erosion. Interestingly as this study has shown that proteolytic activity is present in the saliva of healthy controls this suggests these changes may occur before overt signs of dental erosion are apparent. A longitudinal study is required to assess this relationship, and to investigate the use of salivary protein biomarkers in dental erosion diagnostics.

As this is the first study to report altered diversity in the salivary microbiome of individuals with dental erosion, further research is required to elucidate pathophysiology. There are a number of hypotheses that may explain this reduced microbial diversity. The 16S rRNA gene sequencing data and the mRNA sequencing data both demonstrate the increased abundance and activity of acid-tolerant bacteria in the dental erosion sample group, including multiple *Actinomyces species*, *Lactobacillus species*,* Streptococcus mutans*, and *Veillonella species* [43]. None of the participants had active caries. Although some of these species, particularly *S. mutans*, were present in very low abundance, their significant increase may still be biologically significant [44]. Individuals with dental erosion have been shown to have increased consumption of dietary acids [45–47] and longer periods of reduced pH in the oral cavity post-consumption [24], and/or gastro-oesophageal reflux disorder (GORD), which decreases the pH in the mouth to approximately 4.9 [48]. These factors promote favourable conditions for erosion and dissolution of the enamel surface. This is the opposite of dental caries, where consistent ingestion of dietary carbohydrates leads to decreased pH in the mouth over an increased period, which in turn results in dysbiosis of the oral microbiota and leads to the proliferation of endogenous aciduric bacteria, such as *Streptococcus mutans*, which causes damage to the dental tissues [49]. Therefore, the intermittent acidity experienced in the oral cavity by oral bacteria in dental erosion could select for the increased presence of acid-tolerant bacteria. The mRNA sequencing data echoes this theory as a gene for proton transport (*PMA1/PMA2*) was over-expressed in the erosion group, which suggests that the bacteria present are actively resisting a lower pH environment. However, it must be taken into consideration that some genes had different annotations depending on the program or database used. Future studies should corroborate the function of these genes and confirm the significance of this possible annotation. The increase in free amino acids, including proline and glycine, in erosion participants and the over-expression of genes that theoretically resist lower pH environments is in agreement with work conducted by Edlund et al. 2015. In this study, biofilms that were exposed to glucose experienced a drop in pH to 4.2, and a subsequent increase in free amino acid production in response to this change [50]. A limitation of this study is that data on participant’s dietary habits and experience of GORD were not recorded; therefore, further investigations should include this for more in-depth correlation studies. The pH of the oral cavity was also not investigated in this study, and this will be included in future studies.

Another interesting consideration is that individuals with dental erosion historically have less AEP compared to healthy controls [18]; therefore, the reduced microbial diversity may be due, in part, to a reduction in available binding sites for early colonisers of the oral biofilm. It is thought that the early colonisers of the biofilm have a great impact on how the biofilm will further progress by what adherence molecules they lay down (succinctly reviewed by Nobbs et al., 2011 [51]) and the adhesins that are expressed on the bacterial cell surface, and this can affect the oral health of the individual. Further study is required to investigate these theories. The AEP was not assessed in this study along with more localized areas of erosion, so future work should include microbiome analysis of plaque samples and protein concentration and proteomics of the AEP. As this was a preliminary study, the small sample size is a limiting factor here and future studies should build upon this by increasing the sample size.

## Conclusions

This study of individuals with and without dental erosion has demonstrated several significant findings using a multi-omics approach. We have shown that dental erosion is correlated with increased salivary proteolytic activity, although this is not causal, but more likely an association. This relationship has been shown via salivary proteomics, metabolomics, and mRNA sequencing of the salivary microbiome. We have shown further through 16S rRNA gene sequencing and metatranscriptomic sequencing that the salivary microbiome composition and its activity is different between the two groups, with dental erosion participants exhibiting a less diverse microbiome—this is the first study of this kind to report this.

## Supplementary Information

**Additional file 1:** **Figure S1-5.**

## Data Availability

The mass spectrometry proteomics data have been deposited to the ProteomeXchange Consortium via the PRIDE 27 partner repository with the dataset identifier PXD035565 and 10.6019/PXD035565. The NMR data is available at the NIH Common Fund’s National Metabolomics Data Repository (NMDR) website, the Metabolomics Workbench, https://www.metabolomicsworkbench.org, where it has been assigned Project ID PR001447. The data can be accessed directly via its Project DOI: 10.21228/M8PH7X. The 16S rRNA gene sequencing and mRNA gene sequencing raw sequence reads (fastq) have been deposited in the NCBI Sequence Read Archive with the BioProject ID PRJNA866346.
