# Supplementary figures and images for: Novel bacterial proteolytic and metabolic activity associated with dental erosion-induced oral dysbiosis
Source: Microbiome. 2023 Mar 31;11:69. doi: 10.1186/s40168-023-01514-0 (PMC10064782; doi:10.1186/s40168-023-01514-0)

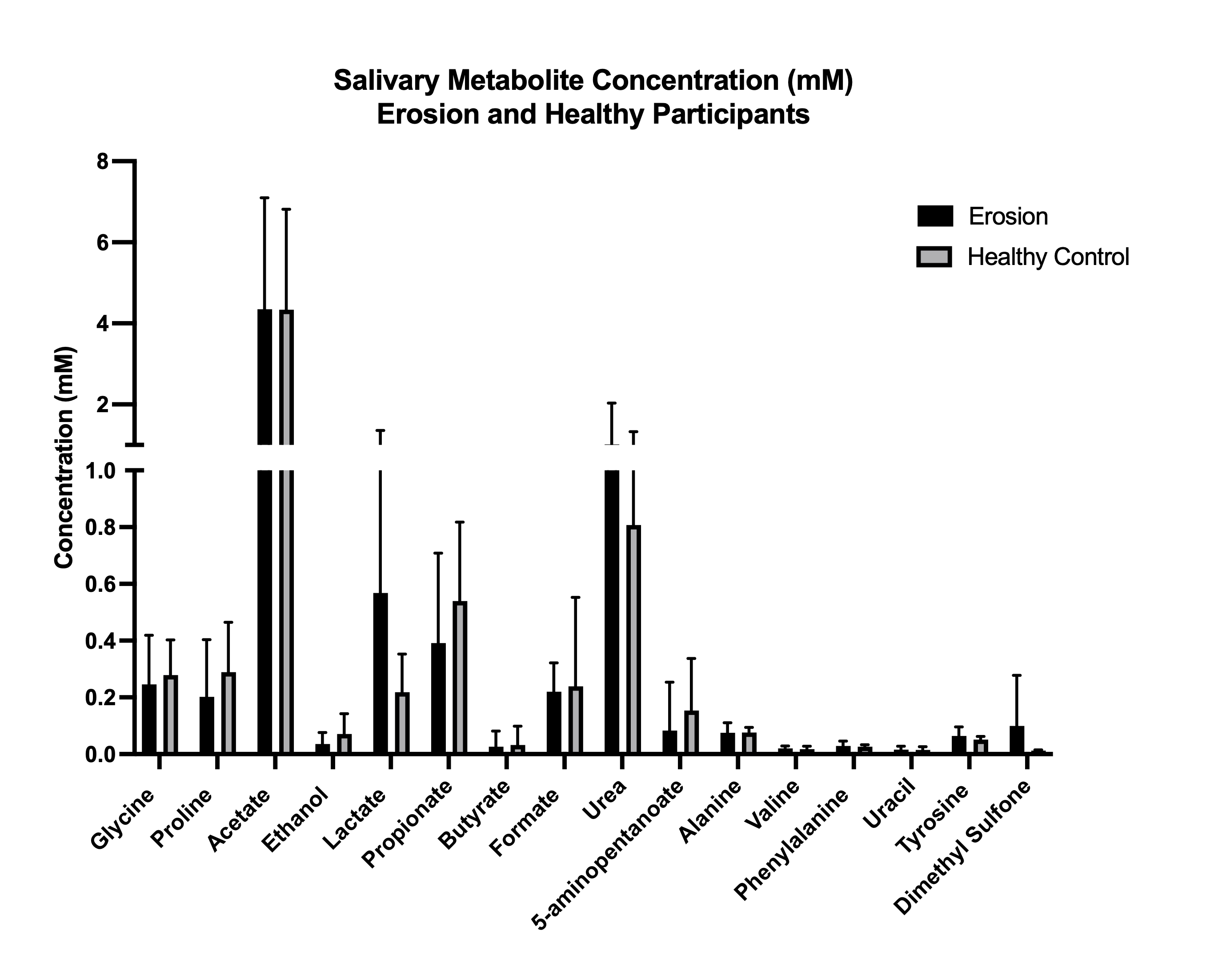

Supplement: Supplementary file 2 — Additional file 1: Figure S1-5. [file 40168_2023_1514_MOESM1_ESM.zip › supplemental figure 4.jpg]

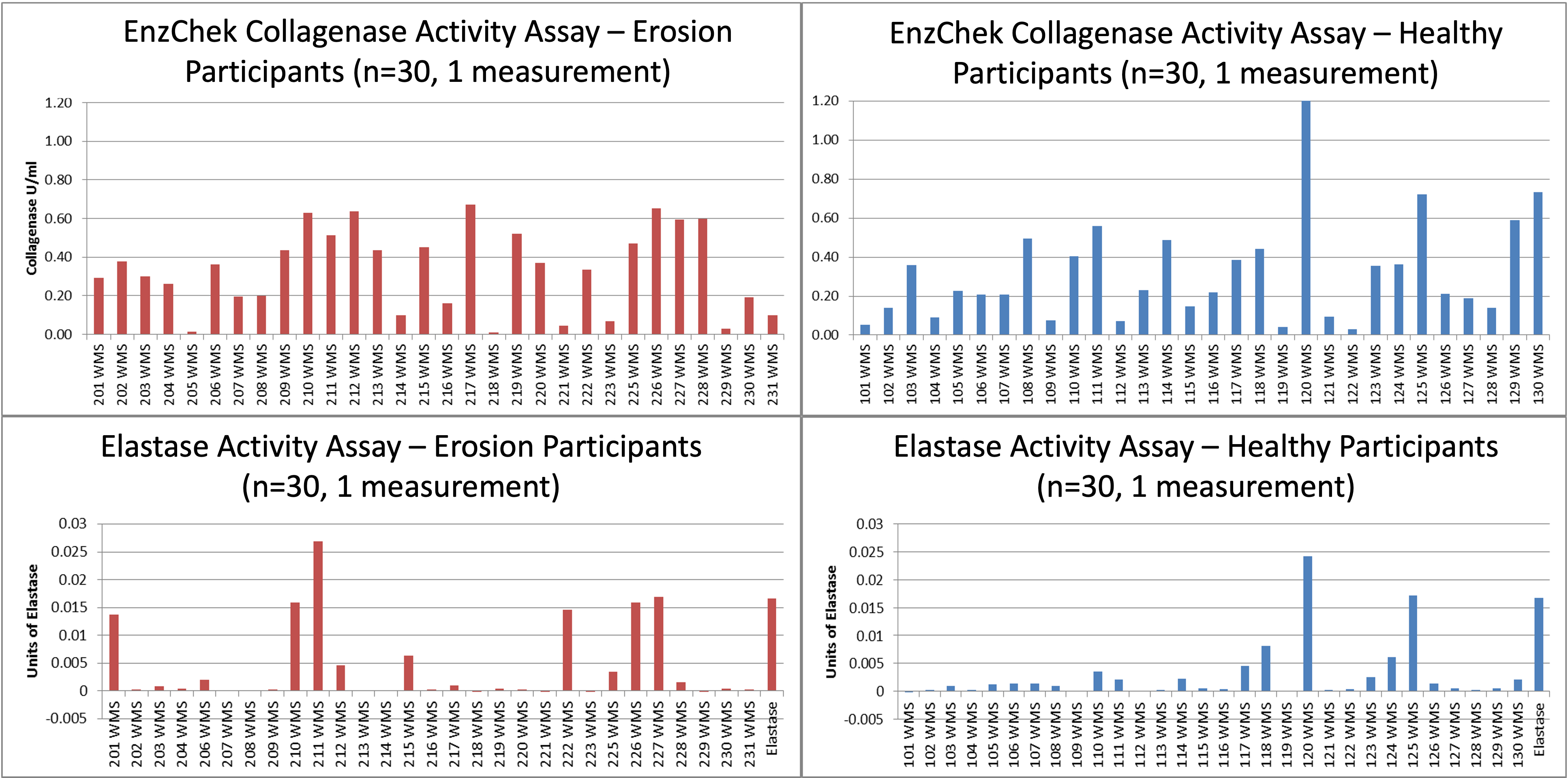

Supplement: Supplementary file 2 — Additional file 1: Figure S1-5. [file 40168_2023_1514_MOESM1_ESM.zip › supplementary figure 1.jpg]

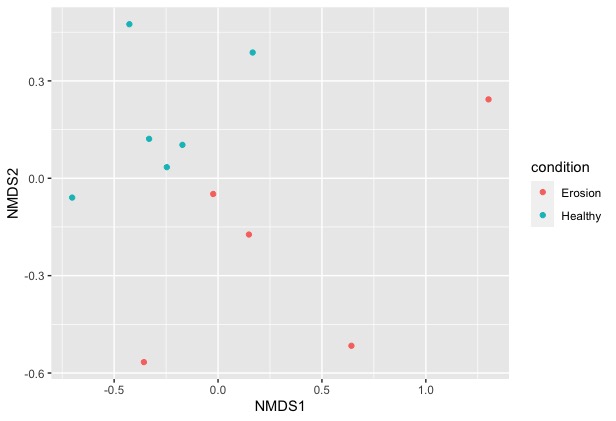

Supplement: Supplementary file 2 — Additional file 1: Figure S1-5. [file 40168_2023_1514_MOESM1_ESM.zip › supplementary figure 2.jpeg]

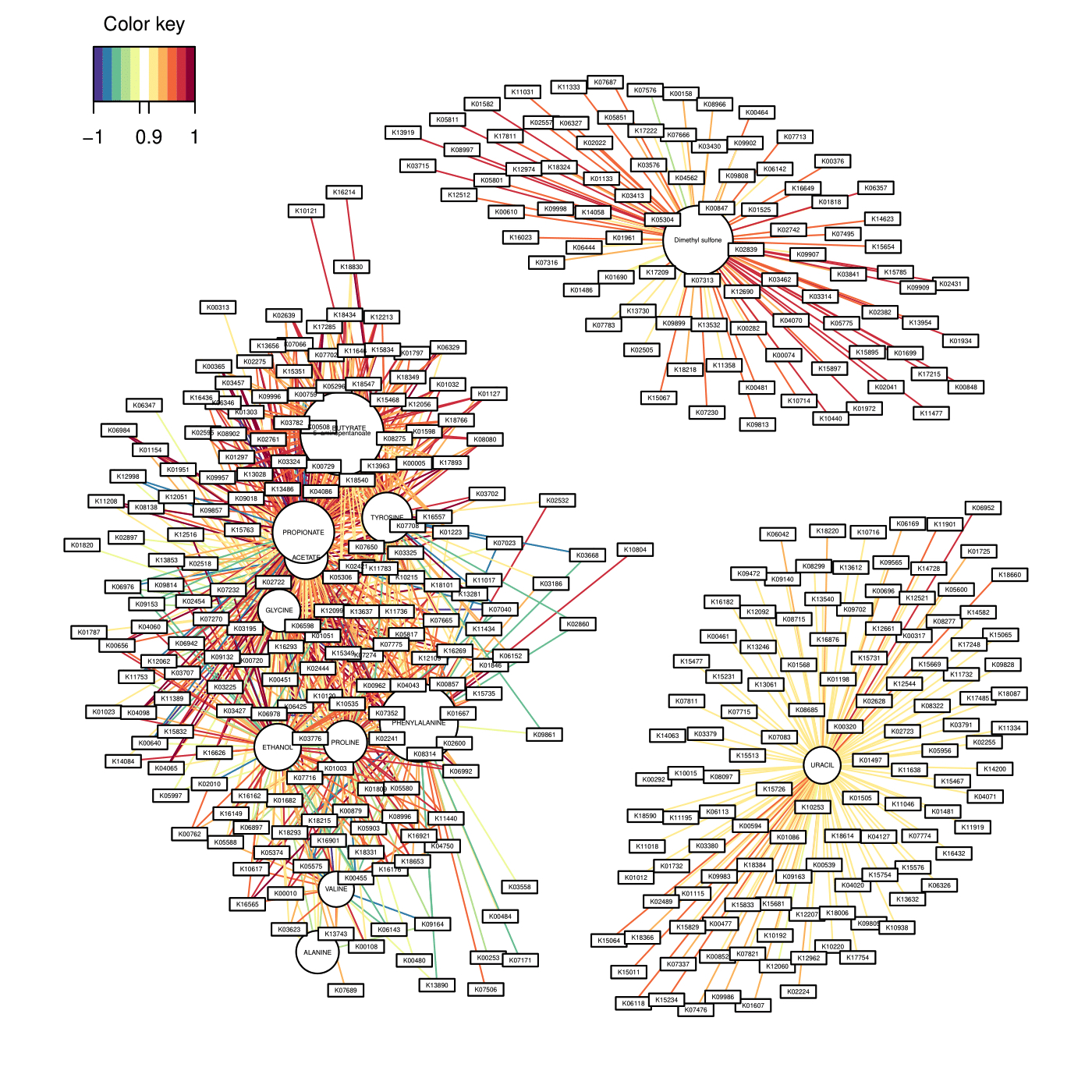

Supplement: Supplementary file 2 — Additional file 1: Figure S1-5. [file 40168_2023_1514_MOESM1_ESM.zip › supplementary figure 5A erosion.jpg]

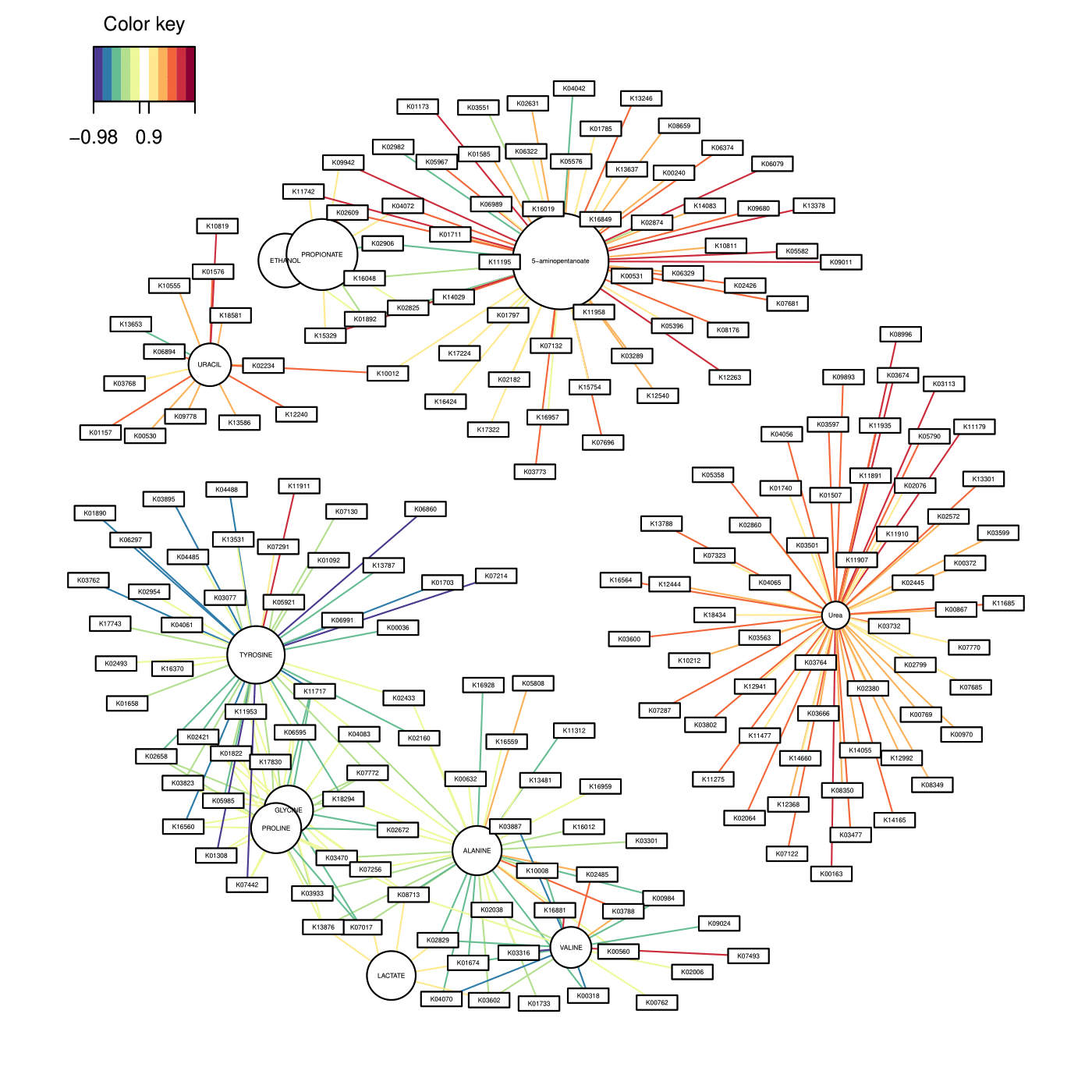

Supplement: Supplementary file 2 — Additional file 1: Figure S1-5. [file 40168_2023_1514_MOESM1_ESM.zip › supplementary figure 5B healthy.jpg]

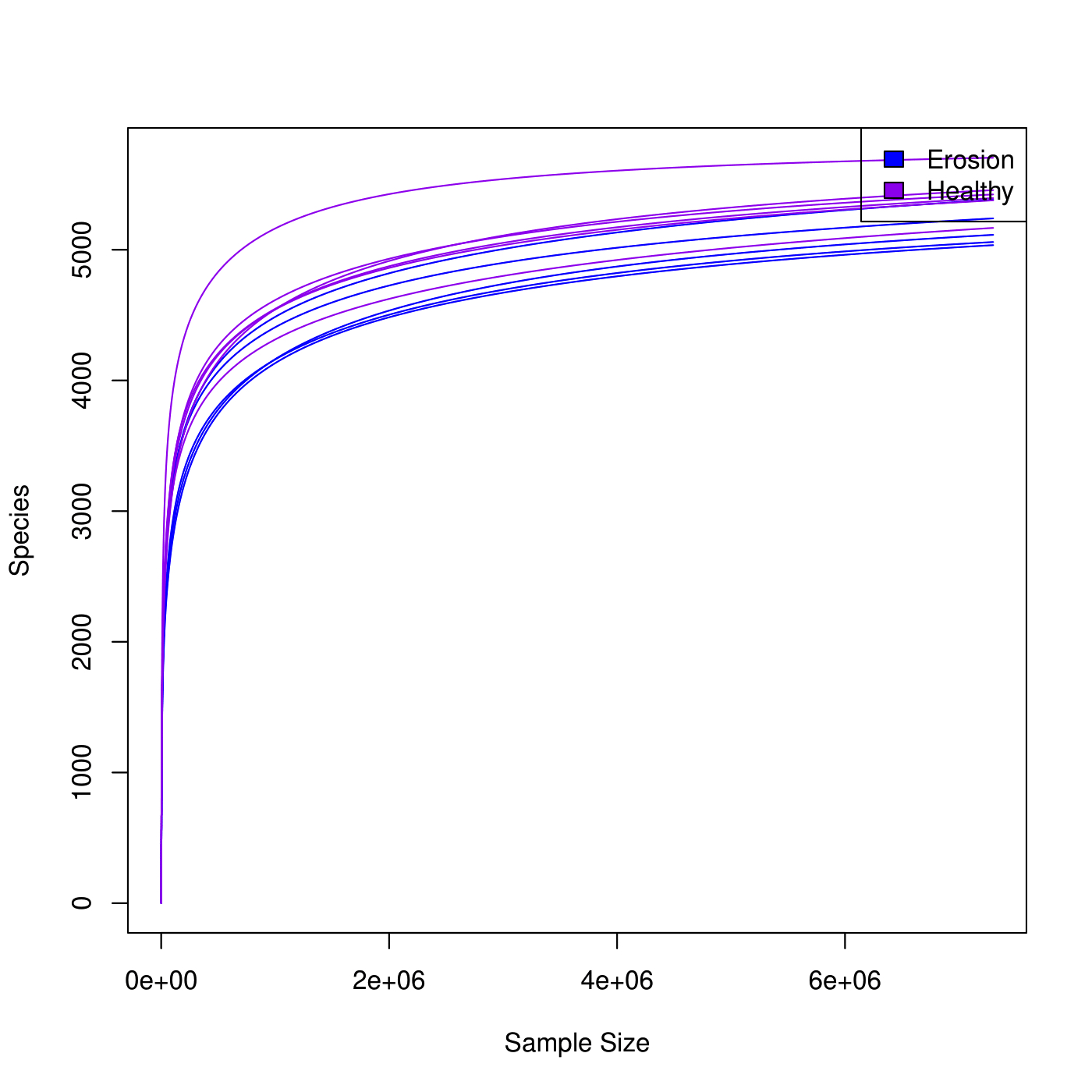

Supplement: Supplementary file 2 — Additional file 1: Figure S1-5. [file 40168_2023_1514_MOESM1_ESM.zip › supplementaryfigure3.jpg]
